# Case Report: Exclusive localization of Leishman-Donovan bodies in neutrophils on peripheral blood smear in a patient with systemic lupus erythematosus

**DOI:** 10.3389/fmed.2025.1693178

**Published:** 2025-12-05

**Authors:** Fucun Ma, Ruixue Zhang, Meilin He, Jia Li, Mingjian Bai, Guowei Liang, Xuekai Liu

**Affiliations:** 1 Department of Clinical Laboratory, Aerospace Center Hospital, Beijing, China; 2 Department of Cell Hematology Laboratory, Aerospace Center Hospital, Beijing, China

**Keywords:** visceral leishmaniasis, systemic lupus erythematosus, Leishman-Donovan bodies, neutrophils, immunosuppression, diagnostic challenges

## Abstract

**Background:**

Visceral leishmaniasis (VL) is a life-threatening parasitic infection transmitted by sand flies. Its clinical manifestations can overlap with those of autoimmune diseases, such as systemic lupus erythematosus (SLE). Traditional diagnostic approaches, like bone marrow aspiration, have limited sensitivity for detecting the parasite in peripheral blood. Immunosuppressed patients often present atypically, further complicating diagnosis. Rarely, *Leishmania* amastigotes (Leishman-Donovan bodies) can be found within neutrophils, which challenges the conventional view that the parasite primarily infects monocytes/macrophages.

**Case presentation:**

A 73-year-old male patient with a history of SLE was on immunosuppressive therapy. The patient presented with persistent pancytopenia and splenomegaly. The prior SLE diagnosis contributed to a delay in recognizing VL. A meticulous examination of the peripheral blood smear first revealed Leishman-Donovan bodies within neutrophils, providing the critical diagnostic clue. This finding prompted further investigations, and the diagnosis was subsequently confirmed by the combination of bone marrow aspiration and serological testing. His immunosuppressed state likely masked typical inflammatory responses and increased his vulnerability to this opportunistic infection, highlighting the difficulty in distinguishing between autoimmune and parasitic diseases.

**Conclusion:**

This case underscores the diagnostic challenge of VL in immunosuppressed patients. Symptom overlap with autoimmune disorders and atypical parasite locations (e.g., within neutrophils) can delay diagnosis. Morphological examination of blood and bone marrow remains crucial for detecting rare pathogens. In endemic areas, clinicians should routinely check for parasites within neutrophils to reduce misdiagnosis. Future studies should explore neutrophil-parasite interactions and improve infection monitoring strategies in immunocompromised hosts.

## Introduction

### Epidemiology and diagnostic challenges

*Leishmania* parasites, which are capable of infecting both humans and animals, are responsible for a disease with extensive global distribution. Visceral leishmaniasis (VL), also referred to as kala-azar, represents a severe manifestation of the disease and is predominantly transmitted through the bites of infected sand flies. Annually, it is estimated that there are between 20,000 and 40,000 new cases of VL worldwide (1). In the absence of treatment, the case fatality rate can surpass 95%. Over 90% of VL cases are concentrated in six countries: India, Bangladesh, Sudan, South Sudan, Ethiopia, and Brazil. In China, *Leishmania* parasites are endemic in mountainous and rural regions of provinces such as Shanxi, Gansu, and Shaanxi, primarily maintained in canine reservoirs, thereby increasing the risk of disease transmission (2). The epidemiology of *Leishmania* infections is shaped by a variety of factors, including environmental changes (such as temperature, precipitation, vegetation, and humidity) and social determinants such as conflict, poverty, and population displacement (3).

### The rarity and diagnostic significance of antineutrophilic parasites

Various methods are employed for the detection of *Leishmania* parasites. Traditional techniques, such as spleen and bone marrow examinations, involve smearing and staining followed by microscopy to identify amastigotes (Leishman–Donovan bodies). While these methods are valuable, they are also invasive. Conversely, peripheral blood smears offer a noninvasive alternative but suffer from notoriously low sensitivity, with detection rates often less than 5%. In contrast, molecular diagnostic techniques, including real-time polymerase chain reaction (real-time PCR) and loop-mediated isothermal amplification (LAMP), provide significantly higher sensitivity, with real-time PCR achieving a sensitivity of 98.7% (4–6). The occurrence of *Leishmania* within neutrophils is infrequent but holds considerable diagnostic significance. Microscopic examination of peripheral blood smears from infected canines can reveal the presence of amastigotes within neutrophils, with species confirmation achievable through molecular analysis. Furthermore, polymerase chain reaction (PCR) can detect *Leishmania* DNA in neutrophils, and immunofluorescence techniques can be utilized to visualize *Leishmania* antigens within these cells.

### Case presentation and diagnostic challenges

This case pertains to a patient with a medical history characterized by xerostomia, dermatological manifestations, and fatigue, culminating in a diagnosis of systemic lupus erythematosus (SLE). The therapeutic regimens included glucocorticoids and immunosuppressive agents. Although the dermatological symptoms initially responded to symptomatic management, the patient subsequently exhibited persistent pancytopenia and progressive splenomegaly. Ultimately, the identification of Leishman-Donovan bodies within neutrophils in peripheral blood and bone marrow smears, coupled with serological confirmation, established a diagnosis of VL. The distinctiveness of this case is underscored by the following elements: (1) Symptom overlap: The hepatosplenomegaly and pancytopenia associated with VL closely resemble the hematological manifestations of SLE, posing a risk of misattribution to SLE exacerbations. (2) Complexity of immunosuppression: Prolonged immunosuppressive therapy may heighten vulnerability to parasitic infections such as VL while obscuring typical inflammatory responses, thereby complicating the diagnostic process. (3) Detection of rare pathogens: The diagnosis of VL predominantly depends on pathogen-specific diagnostic tests, including bone marrow smear analysis. Nevertheless, in laboratories where expertise in parasitological morphology is limited, the positivity rate remains exceedingly low. This particular case emphasizes the essential role of thorough examination of peripheral blood smears in the identification of pathogens within neutrophils, underscoring the importance of morphological assessment in atypical cases.

## Case presentation

A 73-year-old male, a long-term resident of a rural area of Beijing without travel history to known endemic regions for VL, was admitted on 20 March 2025. He presented with an 11-month history of intermittent dry mouth, rash, accompanied by 5 months of progressive fatigue and anorexia, and weight loss, with symptoms initially beginning in July 2024.

His medical journey began several months earlier. On 20 September 2024, he presented to our hospital. Laboratory investigations at that time revealed pancytopenia [white blood cell (WBC) count 3.26 × 10^9^/L, hemoglobin (Hb) 96 g/L, platelet (PLT) count 83 × 10^9^/L], splenomegaly, proteinuria, elevated D-dimer, positivity for multiple autoantibodies [including antinuclear (ANA) and anti-double-stranded DNA (anti-dsDNA)], and significant hypergammaglobulinemia. Bone marrow cytology showed hyperplasia with decreased granulocytic and erythroid ratios. Based on these findings, a preliminary diagnosis of SLE and possible primary biliary cholangitis was made.

Treatment for SLE was initiated, including glucocorticoids, mycophenolate mofetil, hydroxychloroquine, belimumab, and hepatoprotective agents. While his dermatological symptoms improved, pancytopenia persisted, and he was switched to oral cyclosporine in December 2024. Approximately five months prior to the March 2025 readmission, the patient self-discontinued all the medications, resulting in worsened anorexia and fatigue and necessitating a reduction of prednisone to 5 mg/day.

The patient’s condition culminated in readmission on 20 March 2025, due to worsening cytopenia (WBC 1.17 × 10^9^/L, Hb 88 g/L, PLT 88 × 10^9^/L), proteinuria, and gross hematuria. Despite treatment, his pancytopenia progressed and was accompanied by coagulopathy and hypoalbuminemia.

**FIGURE 1:**
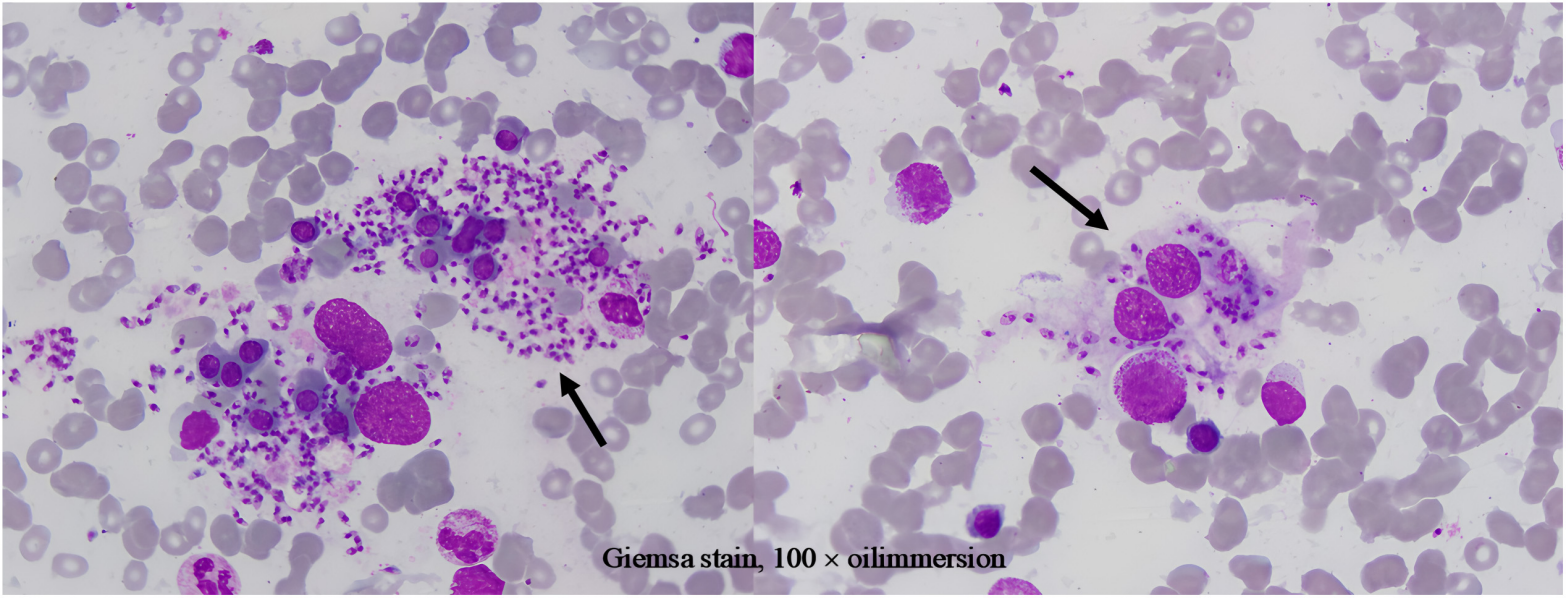
Leishman-Donovan bodies within macrophages on bone marrow smear (Giemsa stain, 100 × oil immersion).

**FIGURE 2:**
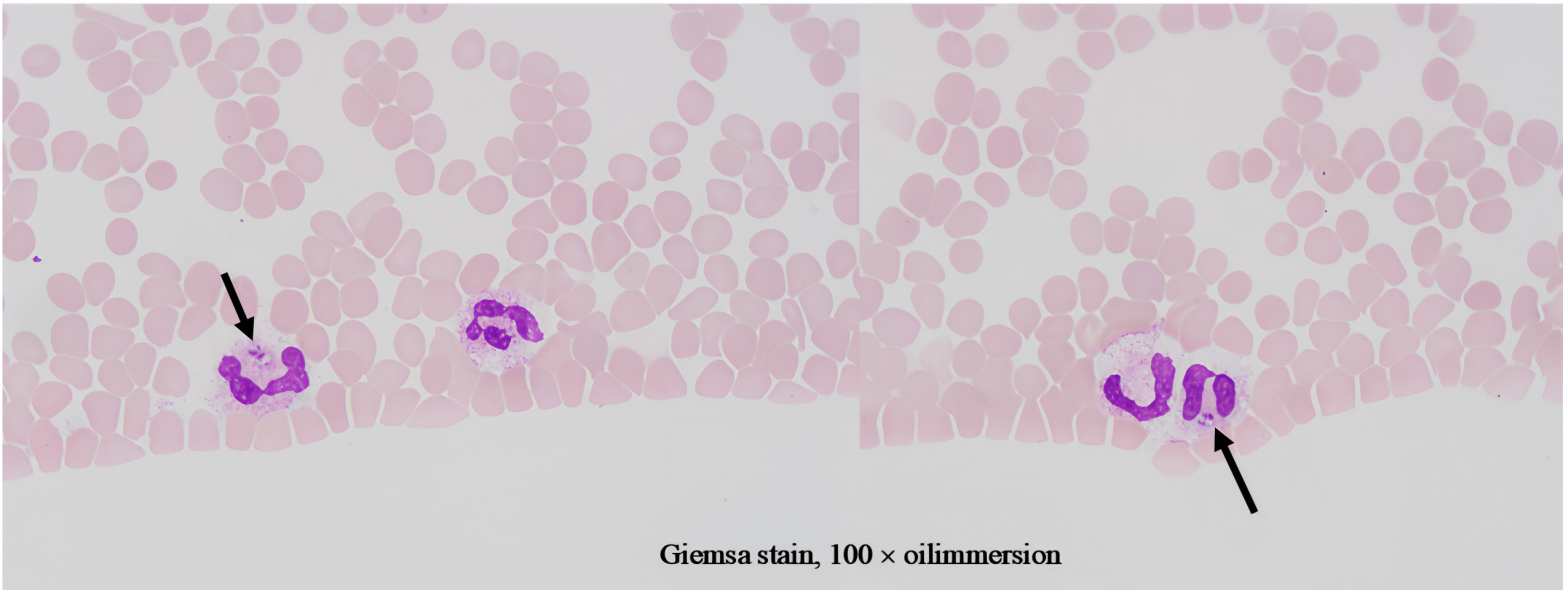
Leishman-Donovan bodies within neutrophils on blood smears (Giemsa stain, 100 × oil immersion). The bodies are approximately 2–3 μm in size, with pale blue cytoplasm containing purplish-red kinetoplasts and nuclei.

Given treatment failure and clinical deterioration, a definitive diagnostic procedure was performed. On 2 April 2025, bone marrow aspiration and biopsy were conducted. While the biopsy later confirmed the presence of numerous Leishman-Donovan bodies within macrophages (Figure 1), the pivotal diagnostic breakthrough occurred upon a subsequent targeted review of the peripheral blood smear. This review, prompted by the clinical dilemma, unequivocally identified multiple Leishman-Donovan bodies within neutrophils (Figure 2). This combined pathological and cytological finding led to the diagnosis of VL on April 2, which was later serologically corroborated by a positive *Leishmania donovani* IgG antibody test on April 10.

The subsequent therapeutic course was challenging. Transferred to a specialized infectious disease department on April 11, the patient began targeted antiparasitic therapy. An initial course of intramuscular sodium stibogluconate (600 mg/day) was halted in early May due to suspected drug-induced pancreatitis. Therapy was switched to amphotericin B but was also discontinued on May 14 due to significant bilateral lower limb edema. Throughout this period, he required ongoing supportive care for persistent complications including hypoalbuminemia, hypokalemia, anemia, and liver dysfunction. His condition eventually stabilized, allowing for discharge on 16 May 2025.

Follow-up and readmission revealed a complex clinical picture. A June 4 follow-up at another hospital showed partial hematological recovery. However, upon readmission on June 19, he reported persistent fatigue and anorexia, with physical examination confirming significant splenomegaly. Labs confirmed persistent pancytopenia and metabolic disturbances. Although *Leishmania donovani* antibodies remained positive, no Leishman-Donovan bodies were found on repeat bone marrow smears. A second attempt with sodium stibogluconate on June 21 was suspended on June 23 due to recurrent elevation of amylase. As of June 24, his condition was relatively stable but antiparasitic treatment remained limited by adverse drug effects. A summary of the key diagnostic and therapeutic timelines is provided in Table 1.

**TABLE 1:** Timeline of overlapping VL infection in a patient with SLE.

Date	2024.09	2024.12	2025.03.20	2025.04.02	2025.04.10	2025.04.11	2025.05.05	2025.05.14	2025.05.16	2025.06.19
Event phase	Initial pres.	Rx change	Re-admission	Dx breakthrough	Confirmation	Tx start (SSG)	Tx change (AmB)	Tx halt (AmB)	Discharge	Re-admission
Diagnosis	SLE	SLE	SLE	Suspected VL	VL	VL	VL	VL	VL	VL
Key findings	Pancytopenia, AutoAb+	–	Deterioration	LD bodies BM and PB	*Leishmania* IgG+	–	–	–	–	Ab+; ↑amylase
Treatment	Immunosuppression	Cyclosporine	↑Immunosuppression	–	–	SSG	Switched to AmB	D/C’d	Supportive	SSG (D/C’d: panc)
Outcome	Partial response	No response	No improvement	Dx confirmed	Dx confirmed	–	Adverse event (panc)	Not tolerated	Stabilized	Tx interrupted

AE, adverse event; AmB, amphotericin B; AutoAb, autoantibody; BM, bone marrow; D/C’d, discontinued; Dx, diagnosis; LD, Leishman-Donovan; PB, peripheral blood; Panc, pancreatitis; Pres., presentation; Rx, prescription/therapy; SLE, systemic lupus erythematosus; SSG, sodium stibogluconate; Tx, treatment; VL, visceral leishmaniasis.

The patient expressed that the persistent fatigue and anorexia severely impacted his daily life. The delay in achieving a definitive diagnosis caused him significant anxiety. He felt relieved upon the confirmation of VL and hoped for effective treatment to restore his health.

## Discussion

Our case illustrates the profound diagnostic challenge of VL in non-endemic areas, a challenge compounded by its mimicry of SLE and, as we report, the atypical intraneutrophilic localization of the parasite. This case exemplifies this diagnostic dilemma, where the initial presentation closely mimicked SLE, leading to a delay in identifying the underlying parasitic infection. The primary and most pivotal morphological finding in this case was the identification of Leishman-Donovan bodies within neutrophils on a routine peripheral blood smear, which immediately redirected the diagnostic trajectory. While the bone marrow examination provided essential confirmatory evidence, it was the peripheral blood smear that served as the initial and decisive diagnostic tool. This rare occurrence not only underscores the critical role of morphological vigilance but also prompts a re-evaluation of conventional diagnostic paradigms in immunocompromised hosts.

This diagnostic challenge is compounded by the evolving epidemiology of VL in China. Between 2017 and 2021, over 1,000 cases were reported nationally, with a notable resurgence and shifting geographic patterns (7). Typically, VL is endemic in provinces like Shanxi and Shaanxi, transmitted via sandfly bites, and presents with nonspecific symptoms like splenomegaly and fever, increasing misdiagnosis risk. Our patient, however, was a long-term resident of Beijing—a non-endemic urban area. This geographical background became a key factor predisposing to “anchoring bias,” as it effectively ruled out VL from the initial differential diagnosis. This stark contrast between his geographic origin and the classic epidemiological picture significantly contributed to the initial low clinical suspicion for VL and the subsequent diagnostic delay.

It is particularly noteworthy that the localization of Leishman-Donovan bodies within different phagocytic cells was a critical diagnostic aspect in this case. Our morphological observations clearly demonstrated the presence of the pathogen within both bone marrow-derived macrophages (Figure 1) and peripheral blood neutrophils (Figure 2). The latter finding is of particular significance, as it directly challenges the conventional paradigm that *Leishmania* primarily infects the mononuclear phagocyte system. This case unambiguously documents parasitism in both cell lineages, with the discovery of intracellular bodies within neutrophils providing a decisive morphological clue for rapid diagnosis. Therefore, in morphological diagnostics, the careful discrimination of the infected cell type is crucial for understanding atypical disease manifestations and preventing misdiagnosis. This case establishes a clear diagnostic hierarchy: the peripheral blood findings critically advanced the diagnosis, while the bone marrow examination served a confirmatory role.

Reflecting on the diagnostic process, it is evident that the high pre-test probability of SLE, supported by classic features and the patient’s residence in the non-endemic area of Beijing, initially precluded consideration of VL. This case, therefore, serves as a critical reminder of “anchoring bias” in clinical reasoning. Notably, at the initial presentation in September 2024, no specific tests for leishmaniasis were performed, as the clinical picture overwhelmingly pointed towards SLE. It highlights an essential lesson: in immunocompromised individuals, the persistence of unexplained core symptoms—such as refractory pancytopenia and progressive splenomegaly in this case—must prompt clinicians to actively broaden their differential diagnosis and search for superimposed opportunistic infections, irrespective of geographic location.

The observation of intraneutrophilic amastigotes is particularly remarkable given the cell’s short lifespan and potent microbicidal arsenal. This paradox can be explained by sophisticated immune evasion strategies employed by *Leishmania*. The parasite not only recruits neutrophils via C-X-C motif chemokine ligand 8 (CXCL8) induction but also manipulates phagosomal maturation to avoid exposure to lethal granules and actively suppresses host defenses by inhibiting reactive oxygen species (ROS) production via mevalonate kinase and degrading neutrophil extracellular traps (NETs) with 3’-nucleotidase/nuclease (8–11). Thus, our finding likely captures a critical transitional phase in the parasite’s life cycle, where neutrophils are exploited as temporary “stealth reservoirs,” facilitating subsequent macrophage invasion under the radar of host immunity. This “immune buffering strategy” transforms a biological curiosity into a phenomenon with direct diagnostic relevance.

The interaction between *Leishmania* and neutrophils remains complex and context-dependent, with studies suggesting roles in both controlling early infection and promoting parasite dissemination (12–14). Despite these debates, our clinical experience unequivocally demonstrates the diagnostic utility of scrutinizing neutrophils. While molecular techniques like kinetoplast DNA (kDNA)-PCR offer superior sensitivity, they are not universally accessible. Our experience underscores that meticulous microscopic examination of blood smears remains an indispensable, cost-effective, and rapid diagnostic tool. It is important to note a limitation of our diagnostic process: specific molecular tests, such as kDNA-PCR, were not performed due to resource constraints in our routine clinical setting. This omission, however, further underscores the critical value of skilled morphological examination. While molecular methods offer superior sensitivity and are highly recommended when available, our case demonstrates that a definitive diagnosis of VL can be achieved through the vigilant microscopic identification of parasites in both bone marrow and, notably, peripheral blood neutrophils. This approach proved vital in our resource-constrained diagnostic scenario. The definitive diagnosis in our patient was established through the combined cytomorphological evidence from bone marrow and peripheral blood, later corroborated serologically.

## Conclusion

In conclusion, this case highlights that VL can emerge as an opportunistic infection in immunocompromised patients, even in non-endemic urban settings. Its clinical mimicry with autoimmune diseases demands a high index of suspicion. The detection of *Leishmania* within neutrophils—a rare but highly significant finding—challenges traditional concepts and offers a crucial diagnostic clue. We advocate for the systematic inclusion of neutrophil examination in the morphological assessment of high-risk patients presenting with unexplained cytopenias and splenomegaly. Future efforts should focus on elucidating the role of neutrophils in human VL and developing better strategies to monitor and balance infection risks in patients requiring immunosuppressive therapy.

## Data Availability

The original contributions presented in this study are included in this article/supplementary material, further inquiries can be directed to the corresponding author.

## References

[1] Kirk M Alvar J Vélez ID Bern C Herrero M Desjeux P Leishmaniasis worldwide and global estimates of its incidence. *PLoS One.* (2012) 7:e35671. 10.1371/journal.pone.0035671 22693548 PMC3365071

[2] Zhou Z Lyu S Zhang Y Li Y Li S Zhou XN. Visceral Leishmaniasis - China, 2015-2019. *China CDC Weekly.* (2020) 2:625–8. 10.46234/ccdcw2020.173 34594724 PMC8392957

[3] Satoskar AR Tarnas MC Abbara A Desai AN Parker DM. Ecological study measuring the association between conflict, environmental factors, and annual global cutaneous and mucocutaneous leishmaniasis incidence (2005–2022). *PLoS Neglect Trop Dis.* (2024) 18:e0012549. 10.1371/journal.pntd.0012549 39325837 PMC11460679

[4] Khosravi S Hejazi SH Hashemzadeh M Eslami G Darani HY. Molecular diagnosis of Old World leishmaniasis: real-time PCR based on tryparedoxin peroxidase gene for the detection and identification of Leishmania spp. *J Vector Borne Dis.* (2012) 49:15–8. 22585237

[5] Pappa SA Kontou PI Bagos PG Braliou GG. Urine-based molecular diagnostic tests for leishmaniasis infection in human and canine populations: a meta-analysis. *Pathogens.* (2021) 10:269. 10.3390/pathogens10030269 33673416 PMC7996766

[6] Rafati S Nzelu CO Kato H Peters NC. Loop-mediated isothermal amplification (LAMP): an advanced molecular point-of-care technique for the detection of Leishmania infection. *PLoS Neglect Trop Dis.* (2019) 13:e0007698. 10.1371/journal.pntd.0007698 31697673 PMC6837287

[7] Guan LR. Current status of kala-azar and vector control in China. *Bull World Health Organization.* (1991) 69:595–601. 1959161 PMC2393248

[8] D’Alessandro S Parapini S Corbett Y Frigerio R Delbue S Modenese A Leishmania promastigotes enhance neutrophil recruitment through the production of CXCL8 by endothelial cells. *Pathogens.* (2021) 10:1380. 10.3390/pathogens10111380 34832536 PMC8623338

[9] Mollinedo F Janssen H de la Iglesia-Vicente J Villa-Pulgarin JA Calafat J. Selective fusion of azurophilic granules with leishmania-containing phagosomes in human neutrophils. *J Biol Chem.* (2010) 285:34528–36. 10.1074/jbc.M110.125302 20801889 PMC2966068

[10] Bamra T Shafi T Das S Kumar M Dikhit MR Kumar A Leishmania donovani Secretory Mevalonate Kinase regulates host immune response and facilitates phagocytosis. *Front Cell Infect Microbiol.* (2021) 11:641985. 10.3389/fcimb.2021.641985 33981628 PMC8110032

[11] Guimarães-Costa AB DeSouza-Vieira TS Paletta-Silva R Freitas-Mesquita AL Meyer-Fernandes JR Saraiva EM 3′-Nucleotidase/nuclease activity allows leishmania parasites to escape killing by neutrophil extracellular traps. *Infect Immun.* (2014) 82:1732–40. 10.1128/iai.01232-13 24516114 PMC3993383

[12] Novais FO Santiago RC Báfica A Khouri R Afonso L Borges VM Neutrophils and macrophages cooperate in host resistance against *Leishmania braziliensis* infection. *J Immunol.* (2009) 183:8088–98. 10.4049/jimmunol.0803720 19923470

[13] Ribeiro-Gomes FL Romano A Lee S Roffê E Peters NC Debrabant A Apoptotic cell clearance of Leishmania major-infected neutrophils by dendritic cells inhibits CD8+ T-cell priming in vitro by Mer tyrosine kinase-dependent signalling. *Cell Death Dis.* (2015) 6:e2018. 10.1038/cddis.2015.351 26658192 PMC4720886

[14] Adem E Cruz Cervera E Yizengaw E Takele Y Shorter S Cotton JA Distinct neutrophil effector functions in response to different isolates of *Leishmania aethiopica*. *Parasites Vectors.* (2024) 17:461. 10.1186/s13071-024-06489-x 39529155 PMC11555981

